# THE RELATIONSHIP BETWEEN AGE DISCRIMINATION AND QUALITY OF LIFE OF DEPENDENT COMMUNITY DWELLING OLDER PERSONS

**DOI:** 10.1093/geroni/igac059.2761

**Published:** 2022-12-20

**Authors:** Felipe Sandoval Garrido

**Affiliations:** University of Tsukuba, TSukuba, Ibaraki, Japan

## Abstract

The objective of this study is to examine the age discrimination suffered by dependent older adults and its effects on their quality of life, using a large representative cohort study. Dependency is defined as having difficulties with activities of daily living, both basic (ADL) and instrumental (IADL). To identify perceived ageism, we used data collected by the English Longitudinal Study of Ageing (ELSA) in 2010–2011 (wave 5) that asked respondents about being discriminated because of their age. Quality of life was measured using the CASP-19 scale. We performed both cross-sectional and longitudinal analyses using the subsequent 2012–2013 (wave 6) and 2018–2019 (wave 9) follow-ups. Multivariable logistic regression analysis was used to estimate the odds ratios of experiencing perceived age discrimination. The results show that a quarter (22%) of all respondents experienced age discrimination. Those suffering from dependency and age discrimination had independently significant lower quality of life scores. Perceived age discrimination was cross-sectionally associated with being male, white, in poor physical and mental health, highly educated, with lower wealth. Longitudinally, with being male (odds ratio -OR-: 1.5), highly educated (OR: 1.3), and poor mental health (OR: 1.7). However, quality of life change was not statistically significant among dependent older persons in subsequent waves. Understanding ageism is important to create policies for future interventions. The present results reinforce the idea of previously documented groups at risk of age discrimination in order to protect them, but also the complex panorama of bio-psychosocial determinants involved in tackling it.

